# Cancer patients’ perspectives and practices regarding pharmacogenomic testing and its cost

**DOI:** 10.3389/fphar.2026.1737926

**Published:** 2026-04-21

**Authors:** Mohammad Dweib, Hussein Hallak, Hani Hour

**Affiliations:** 1 College of Pharmacy and Medical Sciences, Hebron University, Hebron, Palestine; 2 College of Public Health, Al-Quds University, Jerusalem, Palestine; 3 College of Medicine Al-Quds University, Jerusalem, Palestine

**Keywords:** cancer, KAP, Palestine, pharmacogenomics, willingness-to-pay

## Abstract

**Introduction:**

Pharmacogenomics is a cornerstone of precision medicine. Its major aim is to tailor drug therapy based on individual genetic variations which influence drug pharmacokinetics and dynamics. In the field of oncology, pharmacogenomic testing has become an efficient tool for predicting treatment outcomes and avoiding severe adverse reactions. Understanding the perspectives, attitudes, and willingness of cancer patients toward pharmacogenomic testing is critical for successful implementation. Despite that, research in this area is still inadequate in the Middle East.

**Method:**

This cross-sectional study utilized a survey distributed to cancer patients currently receiving chemotherapy at three outpatient oncology clinics in the West Bank, Palestine.

**Results:**

The study included 368 participants with an almost equal gender distribution. Only 19.8% of the participants had heard of pharmacogenomic testing, while 4.9% of them claimed to have performed a pharmacogenomic test. About one-third of participants reported no understanding of pharmacogenomic testing, with another 31% understanding “a little.” Most of the participants (74%, n = 274) were willing to provide a blood sample for the test, and 53% were willing to pay for the test. The mean maximum out-of-pocket amount the participants were willing to pay was 230 NIS, while the mean reasonable price was 1307 NIS.

**Conclusion:**

Across all dimensions, Palestinian patients demonstrated limited awareness but moderate acceptance of pharmacogenomic testing once given some information. Most of the participants expressed their willingness to undergo genetic testing if it could reduce their risk of severe side effects, hospitalizations, or treatment delays.

## Introduction

Pharmacogenomics is a cornerstone of precision medicine (Sadee et al., 2023). Its major aim is to tailor drug therapy based on individual genetic variations which influence drug pharmacokinetics and dynamics (Cecchin and Stocco, 2020). In the field of oncology, pharmacogenomic testing has become an efficient tool for predicting treatment outcomes and avoiding severe adverse reactions (Talebi et al., 2022).

Understanding the perspectives, attitudes, and willingness of cancer patients toward pharmacogenomic testing is critical for successful implementation (Maska et al., 2025). Patient perspectives are critical in shaping the feasibility of pharmacogenomic testing (Melendez et al., 2023). Despite that, research in this area is still inadequate in the Middle East (Jithesh and Scaria, 2017). Factors such as healthcare financing, cultural values, and health education may impact patient decisions (Ibrahim et al., 2025).

Research in Europe and North America has shown that awareness about these tests remains very low, but patients’ interest rises substantially once they receive simple explanations of the nature of the test and the expected benefits. This indicates that communication and education are key determinants of patient’s acceptance (Veilleux and Bouffard, 2019; Zubiaur et al., 2022).

Financial and ethical considerations can also influence patients’ decisions. Studies consistently reported that cost, privacy, and clarity of benefit are the most important factors that affect patients’ willingness to undergo pharmacogenomic testing (Allen et al., 2022). In settings with limited resources or high out-of-pocket health expenditure, these barriers are augmented. Understanding patient attitudes and willingness to pay is therefore a necessity for proper implementation of pharmacogenomic testing (Abushanab et al., 2025).

While several studies have examined patients’ knowledge and perspectives toward pharmacogenomic testing in high-income countries, evidence from low- and middle-income settings is insufficient. According to our knowledge, no study has assessed how Palestinian cancer patients perceive such testing and how financial, cultural, and ethical factors impact their decisions. Consequently, this study aims to assess cancer patients’ awareness, attitudes, and willingness to pay for pharmacogenomic testing in Palestine.

## Methods

### Study design

This cross-sectional study utilized a questionnaire distributed to cancer patients currently treated with chemotherapy at three outpatient oncology clinics in the West Bank, Palestine.

### Population

All cancer patients receiving or will receive chemotherapy at the time of recruitment, meeting all the inclusion criteria and none of the exclusion criteria.

#### Inclusion criteria

- Patient with a confirmed diagnosis of cancer of any type
- Prescribed or will be prescribed chemotherapy
- Adults (≥18 years)
- Able to understand and provide informed consent

#### Exclusion criteria

- Cognitive impairment or severe psychological distress
- Age less than 18 years

### Sample size calculation

The required sample size was estimated with the single-proportion formula for cross-sectional studies, n = Z^2^p(1−p)/d^2^.

A conservative expected proportion of adequate knowledge (pp) of 0.50 was chosen to maximize the precision; the confidence level was set at 95% (Z = 1.96), and the allowable margin of error (dd) at 5%. Substituting these values yielded n = 384.

Because the total number of eligible oncology patients expected to attend the participating clinics during the data-collection period was approximately 1,200, a finite-population correction was applied: nadj = n/[1+(n−1)/N] = 305.

To correct for an anticipated 15% non-response rate, the sample size was inflated to give final target sample of 350 patients.

### Sampling method

Patients were chosen through convenience sampling from oncology outpatient clinics in the three hospitals (Beit Jala, Dura and Al-Ahli).

### Data collection tool

A structured questionnaire that was developed based on an extensive literature review of similar studies (Asiedu et al., 2020; Cuffe et al., 2012; 2014; Gawronski et al., 2023; 2024; Haga et al., 2016; Soueid et al., 2024). The questionnaire was initially constructed in the English language. After that, it was translated into Arabic with the help of an authorized translator specializing in the medical field.

Both Arabic and English versions were reviewed by five experts who suggested minor modifications to some of the questions. The changes were discussed, and minor changes were made before final approval of the questionnaire. The questionnaire consisted of three main domains: knowledge, attitudes, and practices.

The questionnaire was self-administered. If the participant was illiterate or unable to complete it by themselves, a family member assisted the participant.

When the participant expressed low levels of acceptance to the pharmacogenomic test accompanied by low levels of awareness, the data collectors provided a very brief explanation of the nature of the test. The explanation included a standardized, brief description stating that pharmacogenomic testing is a genetic test used to help doctors select the most effective medications and reduce the risk of adverse drug reactions. No additional details regarding cost, accuracy, or clinical outcomes were provided to minimize influence on participants’ responses.

Participants were asked to report the maximum amount they would personally be willing to pay for pharmacogenomic testing and, separately, what they considered to be a reasonable cost for such testing, to distinguish between personal affordability and perceived value.

A pilot study included 32 participants to assess the validity and reliability of the questionnaire. Cronbach’s alpha for the knowledge, attitudes, and practices domains was all above 0.7, indicating acceptable internal consistency.

The full questionnaire is available from the authors upon reasonable request.

### Ethical measures

Ethical approval to perform the study was acquired from the administration of the School of Public Health and Ethical Committee in Al-Quds University (REF #13/24). Consequently, ethical approval was obtained from the Palestinian Ministry of Health. All participants provided informed consent prior to participation.

## Results

### Sociodemographic and clinical characteristics of the participants

The study included 368 participants with an almost equal gender distribution (52.4% female, 47.6% male). The mean age of the participants was 42 years. The most commonly reported marital status was married (38%), followed by single (33%). Secondary education was the most common educational level (32%), and around 21% reported that they were unemployed. Monthly income was mostly in the lower ranges, with 45% earning 1,000–3000 NIS a month.

Breast cancer (31%) and colon cancer (31%) were the most common types of cancer affecting the participants. Stage III was the most commonly reported stage (22%), while 23% could not identify their cancer stage. Chemotherapy was the most common type of treatment (45%).

Sociodemographic and clinical information are shown in Table 1.

**TABLE 1:** Sociodemographic and clinical information of the participants.

Variable	Category	N	Percent
Gender	Female	193	52.4%
	Male	175	47.6%
Marital status	Single	124	33.7%
	Married	141	38.3%
	Divorced	38	10.3%
	Widowed	55	14.9%
	Unknown	10	2.7%
Education	None	14	3.8%
	Primary	81	22.0%
	Secondary	119	32.3%
	University	99	26.9%
	Graduate	50	13.6%
	Other/Missing	5	1.4%
Employment	Full-time	91	24.7%
	Part-time	43	11.7%
	None	79	21.5%
	Retired	41	11.1%
	Housewife	70	19.0%
	Other	37	10.1%
	Missing	7	1.9%
Income	≤1,000	107	29.1%
	1,000–3,000	168	45.7%
	3,000–5,000	63	17.1%
	>5,000	24	6.5%
	Missing	6	1.6%
Family size	1	83	22.6%
	2	88	23.9%
	3–4	89	24.2%
	≥5	100	27.2%
	Missing	8	2.2%
Cancer type	Breast	116	31.5%
	Colon	116	31.5%
	Lung	95	25.8%
	Other	35	9.5%
	Missing	6	1.6%
Cancer stage	Stage 1	72	19.6%
	Stage 2	60	16.3%
	Stage 3	83	22.6%
	Stage 4	59	16.0%
	Don’t know	88	23.9%
	Missing	6	1.6%
Treatment	Chemotherapy	169	45.9%
	Radiotherapy	60	16.3%
	Surgery	76	20.7%
	Targeted	59	16.0%
	Missing	4	1.1%

### Knowledge

**FIGURE 1:**
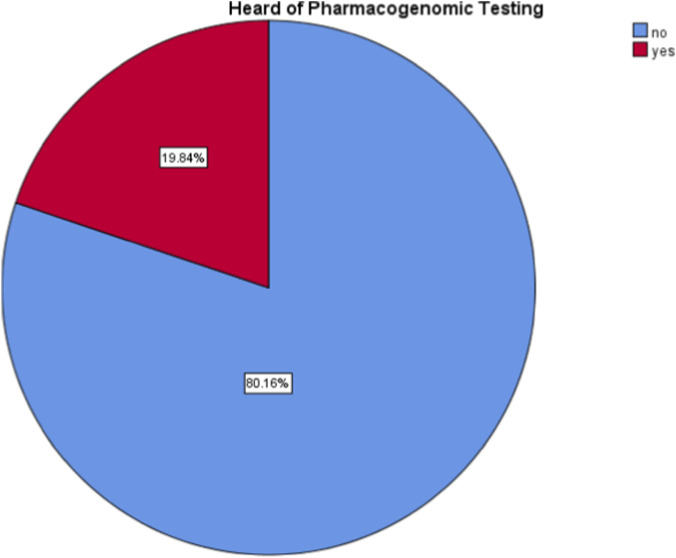
Participants who had heard of pharmacogenomic test.

**FIGURE 2:**
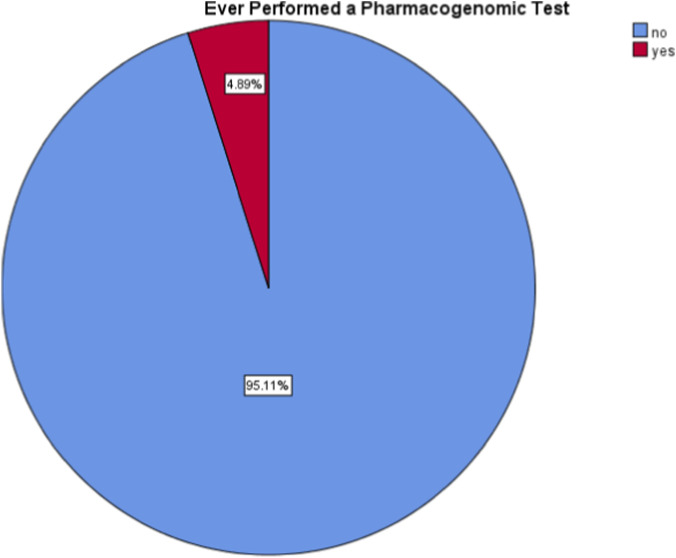
Participants who performed a pharmacogenomic test.

A percentage of 19.8% of the participants had heard of pharmacogenomic testing, while only 4.9% of them claimed to have performed a pharmacogenomic test (Figures 1, 2).

About one-third of participants (33.4%) reported no understanding of pharmacogenomic testing, with another 31% understanding “a little.” One-fifth agreed, and 17.9% strongly agreed that testing can help doctors choose the best treatment. Similar results were observed for the perceived accuracy stated by the participants. These data are shown in Table 2.

**TABLE 2:** Participants’ understanding of pharmacogenomics and their attitudes towards their importance and accuracy.

Question	Category	Count	N %
Understanding pharmacogenomics	Don’t understand	123	33.4%
	Understand a little	115	31.3%
	Somewhat understand	56	15.2%
	Understand well	55	14.9%
	Understand very well	19	5.2%
Pharmacogenomics helps doctors	Strongly disagree	75	20.4%
	Disagree	87	23.6%
	Neutral	57	15.5%
	Agree	83	22.6%
	Strongly agree	66	17.9%
Pharmacogenomics accuracy	Strongly disagree	77	20.9%
	Disagree	83	22.6%
	Neutral	46	12.5%
	Agree	93	25.3%
	Strongly agree	69	18.8%

### Attitudes and preferences

**FIGURE 3:**
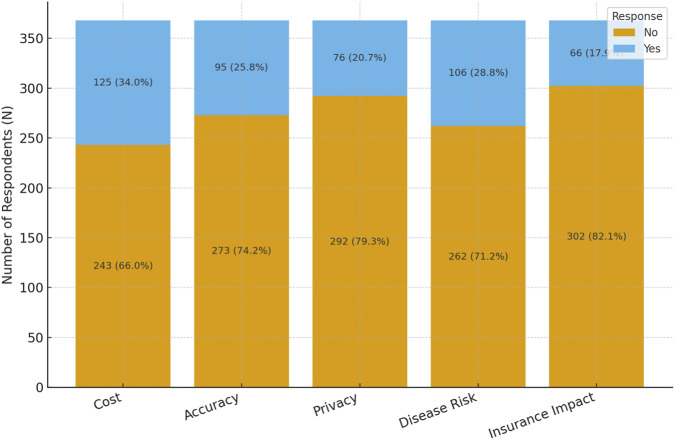
Main concerns about pharmacogenomic testing.

Forty percent of participants were not concerned about insurance impact of the test, and around 40% wanted to be involved in the testing decision. A similar percentage (42%) would be more likely to test if recommended by their physician. Around two-thirds of participants preferred test results to predict both treatment effectiveness and side-effects. The attitudes and preferences of participants are shown in Table 3 while the main concerns of participants are shown in Figure 3.

**TABLE 3:** Attitudes and preferences of participants regarding pharmacogenomic testing.

Question	Response category	Count	Column N %
Do you think pharmacogenomic testing can help avoid side effects from medications?	Strongly disagree	71	19.3%
	Disagree	94	25.5%
	Neutral	52	14.1%
	Agree	83	22.6%
	Strongly agree	68	18.5%
Are you worried about pharmacogenomic testing identifying another disease?	No	149	40.5%
	Yes	155	42.1%
	Not sure	64	17.4%
Do you want to make sure no inherited diseases are revealed by pharmacogenomic testing?	No	157	42.7%
	Yes	147	39.9%
	Not sure	64	17.4%
Are you concerned that pharmacogenomic testing results may affect your health insurance coverage?	No	150	40.8%
	Yes	159	43.2%
	Not sure	59	16.0%
Do you want to be involved in the decision to undergo pharmacogenomic testing?	No	162	44.0%
	Yes	148	40.2%
	Not sure	58	15.8%
Would you undergo pharmacogenomic testing if your doctor recommended it?	No	151	41.0%
	Yes	158	42.9%
	Not sure	59	16.0%
Which results from pharmacogenomic testing would you prefer to receive?	Effectiveness of the medicine	27	7.3%
	Possible side effects	39	10.6%
	Both	242	65.8%
	Not sure	60	16.3%
What is the maximum time you are willing to wait for pharmacogenomic test results?	Same day	84	22.8%
	1–3 days	81	22.0%
	4–7 days	70	19.0%
	1–2 weeks	66	17.9%
	More than 2 weeks	67	18.2%

### Practices and willingness to pay

Most of the participants (74%) were willing to provide a blood sample for the test. Willingness to pay was almost evenly split between those who are willing and those who are not willing to pay for the test (53% willing to pay). The government (34%) and donors (22%) were seen as the main entities responsible for covering the cost of the test. The majority of the participants (86%) did not agree to co-pay for the test. Most of the participants stated that decision-making should be mainly shared between the participant and doctor (71%). Table 4 shows the details of the practices and willingness to pay.

**TABLE 4:** Practices and willingness to pay.

Question	Response category	Count (N)	%
Would you be willing to provide a blood sample for pharmacogenomic testing?	No	94	25.5%
	Yes	274	74.5%
Are you willing to pay for pharmacogenomic testing?	No	172	46.7%
	Yes	196	53.3%
Who should pay for pharmacogenomic testing?	Patient	20	5.4%
	Hospital	44	12.0%
	Government	127	34.5%
	Insurance	55	14.9%
	Donor organizations	81	22.0%
	Don’t know	41	11.1%
Would you agree to co-pay for pharmacogenomic testing?	No	317	86.1%
	Yes	51	13.9%
Who should make the decision to undergo pharmacogenomic testing?	Myself	27	7.3%
	Doctor	39	10.6%
	Both patient and doctor	263	71.5%
	Someone else	39	10.6%

### Amount willing to pay and reasonable price

The mean maximum out-of-pocket amount the participants were willing to pay was 230 NIS, while the mean reasonable price was 1,307 NIS. The details are shown in Table 5.

**TABLE 5:** Maximum out-of-pocket amount and reasonable price.

Question	N	Minimum	Maximum	Mean	Std. Deviation
What is the maximum amount you are willing to pay out-of-pocket for pharmacogenomic testing (in NIS)?	368	0	1,000	230.30	153.15
What do you consider a reasonable cost for pharmacogenomic testing (in NIS)?	368	0	3,000	1,307.88	779.80

Understanding of pharmacogenomic testing differed across demographic groups. Participants with no education were more concentrated in the “do not understand” category (64.3%), while those with primary education were more often in “a little” (42.0%). By contrast, university and graduate participants displayed more balanced distributions, with greater shares in the “somewhat,” “well,” and “very well” categories.

Full-time workers showed more spread across all levels of understanding, with around one-third each in “do not understand” and “a little,” but higher proportions in the “somewhat” and “well” categories compared with unemployed and part-time workers. Retired and housewives tended to cluster in lower understanding levels, though a small share reached “well” or “very well.”

Low-income groups (≤1,000 and 1,000–3,000 NIS) were mainly in the “do not understand” and “a little” categories (over 60% combined). Higher-income groups (>3,000 NIS) showed a more even distribution, with modestly larger shares in the “well” and “very well” categories.

No statistically significant differences in knowledge were seen between the categories of these variables. Full details of these relationships are presented in Table 6.

**TABLE 6:** Relationship between understanding pharmacogenomics and education, employment and income.

Demographic variable	Category	Don’t understand	Understand a little	Somewhat	Well	Very well
Education	None	9 (64.3%)	3 (21.4%)	1 (7.1%)	1 (7.1%)	0 (0.0%)
	Primary	26 (32.1%)	34 (42.0%)	6 (7.4%)	9 (11.1%)	6 (7.4%)
	Secondary	39 (32.8%)	29 (24.4%)	21 (17.6%)	25 (21.0%)	5 (4.2%)
	University	31 (31.3%)	34 (34.3%)	19 (19.2%)	13 (13.1%)	2 (2.0%)
	Graduate	17 (34.0%)	13 (26.0%)	8 (16.0%)	6 (12.0%)	6 (12.0%)
	Missing	1 (20.0%)	2 (40.0%)	1 (20.0%)	1 (20.0%)	0 (0.0%)
Employment	Full-time	31 (34.1%)	28 (30.8%)	13 (14.3%)	15 (16.5%)	4 (4.4%)
	Part-time	18 (41.9%)	11 (25.6%)	6 (14.0%)	5 (11.6%)	3 (7.0%)
	None	21 (26.6%)	24 (30.4%)	16 (20.3%)	14 (17.7%)	4 (5.1%)
	Retired	13 (31.7%)	17 (41.5%)	5 (12.2%)	3 (7.3%)	3 (7.3%)
	Housewife	25 (35.7%)	21 (30.0%)	9 (12.9%)	12 (17.1%)	3 (4.3%)
	Other	12 (32.4%)	12 (32.4%)	7 (18.9%)	5 (13.5%)	1 (2.7%)
	Missing	3 (42.9%)	2 (28.6%)	0 (0.0%)	1 (14.3%)	1 (14.3%)
Income	≤1,000 NIS	36 (33.6%)	33 (30.8%)	15 (14.0%)	21 (19.6%)	2 (1.9%)
	1,000–3,000 NIS	57 (33.9%)	52 (31.0%)	31 (18.5%)	17 (10.1%)	11 (6.5%)
	3,000–5,000 NIS	21 (33.3%)	20 (31.7%)	6 (9.5%)	12 (19.0%)	4 (6.3%)
	>5,000 NIS	7 (29.2%)	8 (33.3%)	3 (12.5%)	5 (20.8%)	1 (4.2%)
	Missing	2 (33.3%)	2 (33.3%)	1 (16.7%)	0 (0.0%)	1 (16.7%)

No statistically significant association between monthly income and willingness to pay for pharmacogenomic testing (p > 0.05). Willingness to pay for pharmacogenomic testing was highest among participants with middle incomes (1,000–5,000 NIS), while both the lowest (≤1,000 NIS) and highest (>5,000 NIS) income groups showed lower and nearly equal proportions of willingness and unwillingness. Other details of this relationship are shown in Table 7.

**TABLE 7:** Relationship between income and willingness to pay.

Income (NIS)	Not willing to pay	%	Willing to pay	%
≤1,000	50	29.1%	57	29.1%
1,000–3,000	78	45.3%	90	45.9%
3,000–5,000	25	14.5%	38	19.4%
>5,000	13	7.6%	11	5.6%
Missing	6	3.5%	0	0.0%

Chi square, p > 0.05.

When stratified by age, no statistically significant differences were observed in participants’ understanding of pharmacogenomic testing (p > 0.05). Across all age groups, the largest proportion of respondents reported “I do not understand,” ranging from 28.3% in older adults to 38.5% in middle-aged adults. Younger adults showed slightly higher percentages in the “understand a little” category (33.1%), while older adults had marginally more respondents in the “well” and “very well” categories (16.7% and 6.7%, respectively). Overall, limited understanding was the predominant pattern across all age groups. These differences are shown in Table 8.

**TABLE 8:** Relationship between age and understanding of pharmacogenomic testing.

Age category	Don’t understand	Understand a little	Somewhat	Well	Very well
Young adult (18–39)	49 (30.6%)	53 (33.1%)	27 (16.9%)	24 (15.0%)	7 (4.4%)
Middle-aged adult (40–59)	57 (38.5%)	42 (28.4%)	20 (13.5%)	21 (14.2%)	8 (5.4%)
Older adult (≥60)	17 (28.3%)	20 (33.3%)	9 (15.0%)	10 (16.7%)	4 (6.7%)

Chi square, p > 0.05.

Participants who strongly agree or agree that pharmacogenomic testing is accurate were more likely to give a blood sample for the test and to pay for the test compared to patients who strongly disagree or disagree. Details are shown in Table 9.

**TABLE 9:** Relationship between thinking that pharmacogenomic tests are accurate and willingness to provide blood sample and willingness to pay.

Perceived accuracy of pharmacogenomics	Blood sample – No	%	Blood sample – Yes	%	Willing to pay – No	%	Willing to pay – Yes	%
Strongly disagree	18	19.1%	59	21.5%	37	21.5%	40	20.4%
Disagree	25	26.6%	58	21.2%	43	25.0%	40	20.4%
Neutral	10	10.6%	36	13.1%	19	11.0%	27	13.8%
Agree	22	23.4%	71	25.9%	42	24.4%	51	26.0%
Strongly agree	19	20.2%	50	18.2%	31	18.0%	38	19.4%

Chi square, p > 0.05.

As no statistically significant associations were identified, detailed subgroup comparisons are presented for descriptive purposes.

### Multivariate analysis

A multivariable logistic regression analysis was performed to identify factors associated with “willingness to pay for pharmacogenomic testing” and “willingness to provide a blood sample”. The regression models included the following independent factors: age, gender, education, income, understanding of pharmacogenomics, perceived accuracy of pharmacogenomic testing, and willingness to undergo testing if it was recommended by a physician. In the analysis, none of these variables were significantly associated with the participants’ willingness to pay. Similarly, none of the independent factors was significantly associated with willingness to provide a blood sample (although male gender showed a borderline lower likelihood of agreeing to provide a blood sample (OR = 0.62, 95% CI: 0.38–1.02, p = 0.062)).

## Discussion

This study is the first to examine how Palestinian cancer patients view pharmacogenomic testing. The findings show strong acceptance but low awareness. These patterns align with global data and reveal major gaps in local systems.

### Knowledge of pharmacogenomic testing

Only 19.8% of participants had heard of pharmacogenomic testing, and less than 5% had undergone any pharmacogenomic test. About one-third reported no understanding of the nature of this test, while around 20% stated that they know it well enough. These findings are similar to global data: in Saudi Arabia, patient awareness was only about 17% (Sukkarieh et al., 2023) while in the U.S., awareness was around 28% (Haga et al., 2016).

The low levels of knowledge in Palestine therefore are similar to international experience but are more intense because of the lack of structured patient education programs and limited integration of genetic testing into clinical practice (Soueid et al., 2024). Notably, participants with higher education and higher income showed better understanding. This highlights how social disparities may worsen inequities in access to genomic advances (Zhang et al., 2022). Addressing these gaps will require technical adoption in addition to tailored educational interventions to empower patients across different socioeconomic backgrounds.

### Attitudes toward testing

Despite their poor knowledge, patient attitudes were generally positive. About 40% of the participants believed that pharmacogenomic testing can help doctors choose the best treatment, while 44% trusted its accuracy. Similar attitudes were reported in Canada and the USA; around half of patients expressed favorable views once the test was explained (Asiedu et al., 2020).

Remarkably, 42% of the participants indicated that they would undergo testing if it was recommended by their physician. This reflects the great influence of healthcare professionals, as also shown in European studies where physician endorsement was the strongest predictor of uptake (Edris et al., 2022; Truong et al., 2020). For Palestine, this suggests that training oncologists and pharmacists to counsel patients can substantially increase test acceptance within patients. Concerns about insurance discrimination were relatively high (43%) compared to the U.S., where up to 30% worry about this (Lemke et al., 2018), reflecting the stronger role the insurance locally plays. This high level of concern could facilitate implementation, because ethical barriers may be as pressing as financial ones (Lee et al., 2018).

### Preferences and practices

Two-thirds of the participants preferred their pharmacogenomic results to predict both the treatment effectiveness and the risk of side effects, which is consistent with international findings which indicate that patients appreciate information about both efficacy and safety (Magavern et al., 2025; Olson et al., 2017). Around 74% of the participants were willing to provide a blood sample, which is also similar to willingness rates between 65% and 80% in Europe and Asia (Haga et al., 2016).

Decision-making choices showed that 71% of the participants were in favor of sharing the decision-making with their doctor rather than leaving the decision to the doctor alone. This reflects a growing demand for autonomy in a system that has traditionally been doctor-driven. For practice, this indicates that introducing pharmacogenomic testing in Palestine should be accompanied by structured decision-making sharing frameworks to ensure that patients remain active partners in their care.

### Willingness to pay and cost perceptions

More than half of the participants (53%) were willing to pay for the test, with a mean willing to pay (WTP) of around 230 NIS (less than 200 USD) and a perceived “reasonable” cost of about 1,307 NIS (less than 400 USD). These amounts are below the usual cost of pharmacogenomic testing in high-income countries (300–600 USD). US patients reported WTP of 100–400 USD (Bielinski et al., 2017) while in Europe, patients typically expect full coverage by public health systems (Peruzzi et al., 2025).

The large difference between the reported maximum willingness to pay (230 NIS) and the perceived reasonable cost (1,307 NIS) may indicate that participants appreciate the value of pharmacogenomic testing but expect high external financial support. This pattern reflects a preference for donations or publicly funded services rather than direct out-of-pocket payment.

Waiting time for the result was almost distributed evenly between all the periods, so more than 60% of the participants were willing to wait for less than 1 week. As test results usually need a period of about 2 weeks for accurate results, this may represent a potential barrier in settings where testing infrastructure is limited.

In Palestine, 34% expected that the government and 22% donors to cover the cost, while only 5% felt patients should pay for the test. This underscores the reality that financial barriers, rather than attitudinal barriers are the greatest challenge to implementation of the testing process (Abbas et al., 2025; Gibson et al., 2017). From a policy perspective, introducing pharmacogenomic testing will require external funding or integration into government health programs, otherwise uptake will remain lower than expected.

### Sociodemographic differences

Higher education and higher income were associated with better knowledge, while age and employment showed less pronounced effects. These results are consistent with worldwide data, where socioeconomic status is the key predictor of pharmacogenomic knowledge (Asiedu et al., 2020; Chapdelaine et al., 2021). Interestingly, the willingness to pay was not strongly linked to income, indicating that financial limitations are prevalent and not limited to lower-income groups (Bignucolo et al., 2023; Zhang et al., 2022).

Additionally, participants who believed that pharmacogenomic testing was accurate were more likely to agree to give blood samples and to pay for the test. This confirms international findings that trust in medical procedure’s validity is a critical driver of uptake and highlights the importance of clear communication between physicians, healthcare institutions and patients (Marzo et al., 2022; Truong et al., 2020).

### Multivariate analysis

In the multivariable analysis, none of the factors that were examined (sociodemographic or perception-related factors) were independently associated with differences in willingness to pay for pharmacogenomic testing or willingness to provide a blood sample. This indicates that acceptance of pharmacogenomic testing among Palestinian cancer patients could be relatively uniform across different population groups and not driven by specific demographic or knowledge-related characteristics. One possible explanation is that wider systemic factors such as financial constraints and healthcare system structure plays a more important role than individual-related predictors. These findings are consistent with the overall pattern that was observed in this study, where general acceptance was moderate despite limited awareness, indicating that improving accessibility and affordability may be more critical than targeting specific subgroups.

Across all dimensions, Palestinian patients demonstrated limited awareness, but moderate acceptance of pharmacogenomic testing once given some information. This is similar to international experience and highlights education, physician engagement, and financial support as the three drivers for successful implementation of the whole procedure. If these barriers are addressed, pharmacogenomic testing could be integrated into clinical chemotherapeutic practice in Palestine and potentially improve treatment safety and outcomes.

## Conclusion

The study indicated that patients are both aware of and anxious about chemotherapy-related side effects. Most of the participants expressed their willingness to undergo genetic testing if it could reduce their risk of severe side effects, hospitalizations, or treatment delays. Specifically, many of the participants indicated their willingness to pay for these services, although their financial capacity varied widely, and concerns about equitable access were clearly evident. This reinforces the aforementioned issue that while demand and acceptance exist, policy structures and health-financing mechanisms are necessary to ensure that testing does not become a privilege that is available only to wealthier patients.

## Limitations

This study has a few limitations. First, it was performed only in the West Bank, which can limit the generalizability of the findings to other regions such as the Gaza Strip and East Jerusalem, where healthcare systems and socioeconomic conditions may differ. Second, the study included only outpatient cancer patients receiving chemotherapy, excluding inpatient populations and those undergoing other treatment modalities, which may have different perspectives toward pharmacogenomic testing. Third, the use of convenience sampling may introduce selection bias and affect representativeness. Finally, the cross-sectional design precludes establishing causal relationships between knowledge, attitudes, and willingness to pay.

## Data Availability

The raw data supporting the conclusions of this article will be made available by the authors, without undue reservation.
